# Low-Noise Millimeter-Wave Down-Conversion Technology for Chip-Scaled Optical Clocks

**DOI:** 10.3390/s25041041

**Published:** 2025-02-10

**Authors:** Shuai Li, Lulu Yan, Enrang Zheng, Zhijing Du, Jun Ruan, Shougang Zhang

**Affiliations:** 1 School of Electrical and Control Engineering, Shaanxi University of Science and Technology, Xi’an 710021, China; 220611027@sust.edu.cn (S.L.);; 2 National Time Service Center, Chinese Academy of Sciences, Xi’an 710600, China; yanlulu@ntsc.ac.cn (L.Y.);; 3 School of Astronomy and Space Science, University of Chinese Academy of Sciences, Beijing 100049, China; 4 Key Laboratory of Time Reference and Applications, Chinese Academy of Sciences, Xi’an 710600, China

**Keywords:** millimeter-wave, chip-scaled optical clock, down-conversion, regenerative frequency division

## Abstract

This article reports on a millimeter-wave (MM-wave) signal down-conversion system with low phase noise for chip-scaled optical clocks. The system utilizes analog regenerative frequency division, low-noise fractional frequency division, and phase-locked frequency division techniques to down-convert a 100 GHz MM-wave signal to 100 MHz with phase noise of −117 dBc/Hz @100 Hz, −133 dBc/Hz @1 kHz, and 10 MHz with phase noise of −124 dBc/Hz @100 Hz and −143 dBc/Hz @1 kHz. The frequency stability of the signal down-converted to 100 MHz is 5.0 × 10^−15^ @ 1 s and 1.8 × 10^−16^ @ 1000 s, while the frequency stability of the 10 MHz signal is 5.7 × 10^−14^ @ 1 s and 5.9 × 10^−16^ @1000 s, both of which decrease to the 10^−16^ level at 10,000 s. This down-conversion system meets the frequency conversion requirements of state-of-the-art chip-based optical clocks and micro-cavity optical combs.

## 1. Introduction

In recent years, optical clocks, with their ultra-high frequency stability and accuracy, have greatly advanced the fields of fundamental physics research and high-precision measurements [1,2]. With the development and maturity of photonic chip technology, chip-scaled atomic clocks have found more applications in space, aircraft, and vehicles. Chip-based optical clocks, which offer two orders of magnitude better stability than microwave clocks, have attracted considerable interest from many research groups [3,4]. However, the signal output of chip optical clocks is laser signals, which are not compatible with existing time–frequency signals. To bridge this gap and enable compatibility with current time–frequency equipment, MM-wave down-conversion technology has remained one of the key challenges for the development of optical clocks [5,6].

Recent progress has been made by scholars both domestically and internationally in the fields of chip optical clocks and MM-wave down-conversion technology. There are three main methods for millimeter-wave down-conversion: one is the frequency division method based on photonic down-conversion technology [7], the second is the integrated circuit method [8,9], and the third is the regenerative frequency divider method [10]. Dat Pham Tien et al. employed the Mach–Zehnder interferometer method to down-convert a 101 GHz MM-wave signal to 17 GHz [7]. However, their optical down-conversion depends on precise optical path alignment and signal synchronization, and errors in these processes can lead to performance degradation or frequency mismatching. Moreover, the system is highly dependent on the frequency value of the signal before down-conversion, which presents challenges for randomly moving receivers and maintaining optical path alignment, thus limiting the robustness and applicability of the system. The phase noise of their 17 GHz down-converted signal was approximately −110 dBc/Hz at 1 MHz [7] but the stability index was not assessed. Millimeter-wave frequency dividers designed using microelectronic technology are currently limited to experimental research and are not yet suitable for mass production or market deployment [8,9]. The maximum frequency of frequency dividers built with regenerative division technology has not reached 100 GHz [10,11]. Chip-scale optical clocks typically rely on micro-cavity optical combs for optical frequency division. The free spectral range of micro-cavity optical combs typically spans from tens of gigahertz to terahertz, with most of this range falling within the MM-wave frequency band [12,13,14]. Therefore, achieving low-noise down-conversion of MM-wave signals to 100 MHz and 10 MHz frequencies is crucial for advancing the application of chip optical clocks [15,16,17,18,19,20,21]. Existing research on MM-wave down-conversion has mostly focused on communication and radar fields, with less emphasis on the additional frequency stability and phase noise during down-conversion. These technologies often rely on intermediate frequency (IF) reference signal down-mixing, which typically fails to meet the high-precision requirements of optical clock systems [22,23,24]. Thus, researching an efficient, low-noise MM-wave down-conversion technique is of great significance for expanding the applications of chip optical clocks.

This paper designs a MM-wave signal down-conversion system based on low-noise regenerative frequency division technology, which successfully down-converts a high-frequency 100 GHz MM-wave signal to 100 MHz and 10 MHz with high quality. A measurement system was then constructed to measure the frequency stability and phase noise of the down-converted output signals. Experimental results show that the frequency stability of the signal down-converted to 100 MHz is 5.0 × 10^−15^ @ 1 s, 7.1 × 10^−16^ @ 100 s, and 1.8 × 10^−16^ @ 1000 s, with phase noise values of −117 dBc/Hz @100 Hz, −133 dBc/Hz @ 1 kHz, −157 dBc/Hz @ 10 kHz, −154 dBc/Hz @ 100 kHz, and −158 dBc/Hz @1 MHz. For the 10 MHz down-converted signal, the additional frequency stability is 5.7 × 10^−14^ @1 s, 2.1 × 10^−15^ @100 s, and 5.9 × 10^−16^ @ 1000 s, with phase noise values of −124 dBc/Hz @ 100 Hz, −143 dBc/Hz @1 kHz, −169 dBc/Hz @ 10 kHz, −169 dBc/Hz @ 100 kHz, and −166 dBc/Hz @ 1 MHz. This study demonstrates that the proposed MM-wave down-conversion system outperforms traditional down-conversion schemes in terms of noise figure and power consumption. It not only achieves significant improvements in frequency conversion efficiency, noise performance, and stability, but also provides a new solution for the application of chip optical clocks. This research is expected to promote the application of chip optical clocks in high-precision timekeeping, quantum communication, and navigation fields.

## 2. Experiment Setup

**Figure 1:**
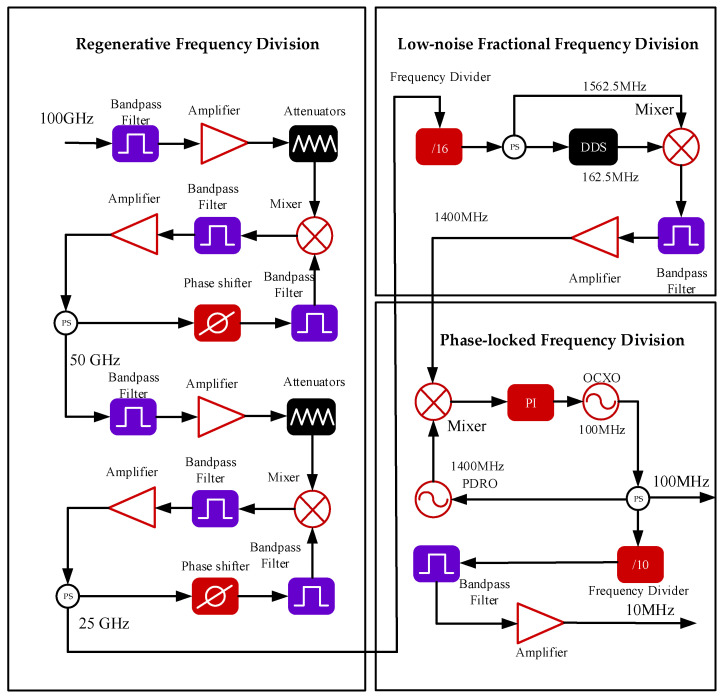
Schematic of the low-noise millimeter-wave down-conversion system. PS: power splitter DDS: direct digital synthesizer; OCXO: oven-controlled crystal oscillator; PDRO: phase-locked dielectric resonator oscillator.

The low-noise MM-wave down-conversion technique incorporates several key technologies: regenerative frequency division, low-noise fractional frequency division, and phase-locked frequency division. The overall schematic of the system is shown in Figure 1.

A 100 GHz signal is processed through two stages of regenerative secondary frequency division, resulting in a down-converted frequency of 25 GHz. The 25 GHz signal is then further divided by a 16-divider to 1562.5 MHz. The divided signal is mixed with a tunable intermediate frequency signal generated by a DDS, producing a 1400 MHz output. After filtering and amplification, the 1400 MHz signal is mixed with the signal from a built-in 1400 MHz phase-locked dielectric resonator oscillator (PDRO) to generate an error signal. This feedback locks the PDRO’s reference 100 MHz voltage-controlled oscillator (VCO), producing a high-precision 100 MHz output. Finally, the high-precision 100 MHz signal is divided by a low-noise 10-divider, resulting in a 10 MHz output.

### 2.1. Regenerative Frequency Division Technology

The key component of the MM-wave down-conversion system is the down-conversion of the 100 GHz MM-wave signal to 25 GHz, as shown in Figure 1. Currently, research on the down-conversion of 100 GHz MM-wave signals is limited, and developing techniques to down-convert the 100 GHz signal to microwave frequencies with high quality is the core focus of this research.

**Figure 2:**
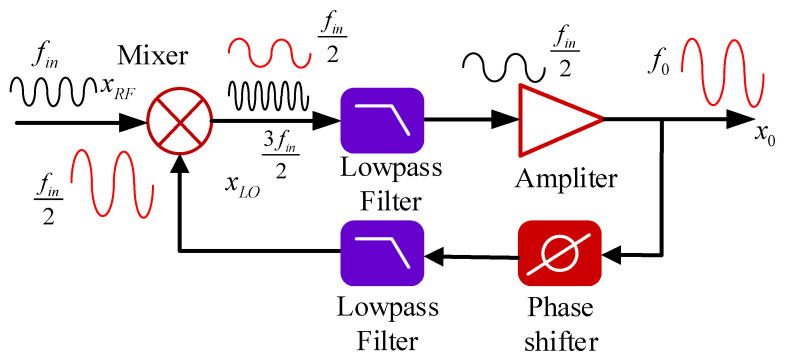
Schematic of regenerative frequency division technology.

The principle of regenerative frequency division is to achieve frequency division of the input signal through a nonlinear element and a feedback loop [25], making it a good choice for high-frequency MM-wave down-conversion. The 100 GHz MM-wave signal enters the regenerative frequency division module through a WR10 waveguide. After filtering and amplification, the 100 GHz signal is fed into the RF port of the mixer. The principle of regenerative frequency division is illustrated in Figure 2.

Let the input frequency of the 100 GHz MM-wave signal be denoted as $f_{in}$, and the output frequency as $f_{0}$. When the signal passes through a nonlinear element, higher-order harmonics are generated. These harmonic frequencies are integer multiples of $f_{in}$. For a divide-by-two system without a frequency multiplier in the loop, stable output requires the condition $f_{0}=f_{in}-f_{LO}$. Because $f_{LO}=f_{0}$ the relationship between $f_{0}$ and $f_{in}$ is given by:

(1) $$f_{0}=\frac{1}{2}f_{in}$$

After the input signal passes through the lowpass filter, the harmonic frequencies are filtered out, leaving only the desired target frequency. The target frequency signal is then amplified to the required power level and fed back into the nonlinear element, i.e., the mixer. To satisfy the closed-loop stability constraint and zero-phase condition, a phase shifter is added to achieve this condition. This process forms a regenerative frequency division system with a stable output frequency signal.

The output frequency of the 100 GHz signal is 50 GHz after the regenerative frequency division technique, and then the 50 GHz signal continues to be regenerated by two divisions to obtain a 25 GHz microwave signal, which is further synthesized to 10 MHz and 100 MHz using low-noise microwave frequency synthesis. The regenerative frequency division technique can be used to improve the phase noise performance in the low-noise frequency division process of MM-wave or microwave signals due to its very low additional phase noise performance in MM-wave or microwave signals [13].

### 2.2. Low-Noise Fractional Frequency Division

The low-noise fractional frequency divider technology is primarily responsible for further frequency division of the 25 GHz signal obtained from the regenerative frequency division technique, ensuring improved frequency stability and reducing phase noise during the down-conversion process. It enhances the accuracy of frequency step resolution, achieving fractional frequency division factors.

The core principle of the low-noise fractional frequency divider technology is to compensate for fractional residual frequencies using direct digital synthesizer (DDS) technology. To overcome DDS quantization errors and associated noise and address the issue of limiting the output signal’s frequency stability to the E-14 @ 1 s level, this paper adopts an approach of reducing the DDS output signal’s weight by a factor of 10. This approach reduces the frequency stability of the DDS output to the E-15 @ 1 s level, thereby achieving low-noise, high-stability fractional frequency division.

The schematic of the low-noise fractional frequency divider module is shown in Figure 1. First, the 25 GHz signal obtained from the regenerative frequency division is divided by a 16-divider to generate a 1562.5 MHz microwave signal. This 1562.5 MHz signal is then split into two paths using a power splitter: one path serves as the reference signal for the AD9912, and the other is prepared to mix with the output signal from the AD9912. The output signal from the AD9912 is adjusted via the PC to produce a difference frequency signal of 162.5 MHz, which is the error between 1562.5 MHz and 1400 MHz This error frequency signal of 162.5 MHz is then mixed with the 1562.5 MHz signal to obtain a stable 1400 MHz output signal.

The choice of low-noise fractional frequency divider technology is based on its short conversion time, reaching nanosecond-level conversion speeds with very fast conversion rates. Furthermore, after mixing the intermediate frequency signal from the AD9912 with the 1562.5 MHz signal, the DDS output frequency is approximately one-tenth of the input signal, with a coupling factor of 10%. Through mixing, the additional Allan deviation stability is optimized by one order of magnitude.

### 2.3. Phase-Locked Frequency Division

Phase-locked frequency division technology locks a 100 MHz OCXO (oven-controlled crystal oscillator) onto the 1400 MHz output signal of the fractional frequency divider, as shown in Figure 1. The process begins with a 1400 MHz PDRO (phase-locked dielectric resonator oscillator), which uses the internal 100 MHz OCXO as its reference driving signal. After filtering and amplifying the 1400 MHz signal from the phase-locked frequency division system, it is mixed with the 1400 MHz signal from the PDRO. The error signal generated by the mixing process is fed back through a loop filter to lock the PDRO’s reference driving signal to the 100 MHz OCXO. The output of this 100 MHz OCXO is the 100 MHz signal generated by the 100 GHz down-conversion. This 100 MHz signal is then further divided by a low-noise 10-divider to obtain the 10 MHz output signal of the 100 GHz down-conversion.

By providing precise frequency control and a feedback mechanism, phase-locked frequency division technology ensures the high-stability and low-noise characteristics of the output signal, making it suitable for applications requiring high signal accuracy. This design not only improves the signal quality but also avoids cumulative frequency offsets caused by multiple frequency conversions, optimizing the overall system performance.

## 3. MM-Wave Down-Conversion System Performance

**Figure 3:**
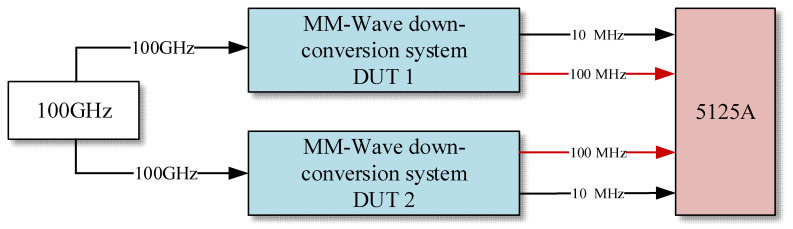
Performance evaluation schematic of mm-wave down-conversion system; DUT: device under test.

To evaluate the performance of the MM-wave down-conversion system, two sets of down-conversion systems with the same structure but independent of each other are constructed. The performance evaluation is carried out by comparing the two sets of down-conversion systems with each other. The block diagram of the evaluation system is shown in Figure 3. The 100 MHz signal and the 10 MHz signal output from the two systems are used as reference and test signals for each other, and the frequency stability and phase noise of the 100 GHz down-conversion output 100 MHz signal and the 10 MHz signal are tested by using the Stability and Phase Noise Analyzer (5125A), respectively. The test interval of the 5125A is 1 s and the bandwidth is 0.5 Hz.

**Figure 4:**
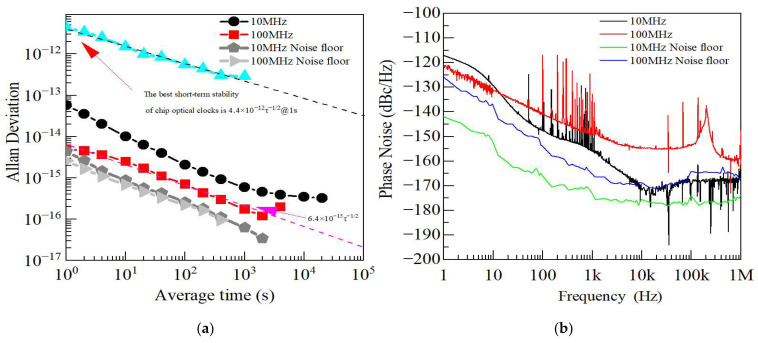
The additional performance test results of 100 MHz and 10 MHz. (**a**) The frequency stability of 100 MHz (solid red square) and 10 MHz (solid red square). The test frequency stability noise floors are shown by the dark-gray pentagon curve (100 MHz) and light-gray triangle curve (10 MHz). (**b**) The additional phase noise of 100 MHz (red curve) and 10 MHz (black curve). The phase noise floors are shown by the blue curve (100 MHz) and green curve (10 MHz).

The test results shown in Figure 4 illustrate the additional frequency stability and phase noise of the low-noise MM-wave down-conversion system, which down-converts the input 100 GHz MM-wave signal to 100 MHz and 10 MHz. Figure 4a shows the test results for frequency stability at 100 MHz and 10 MHz. We set the test interval τ to 1 s with a bandwidth of 0.5 Hz in this case. The frequency stability of 100 MHz is 5.0 × 10^−15^@ 1 s, 7.1 × 10^−16^ @ 100 s, and 1.8 × 10^−16^ @ 1000 s, while the frequency stability of 10 MHz is 5.7 × 10^−14^ @1 s, 2.1 × 10^−15^ @ 100 s, and 5.9 × 10^−16^ @ 1000 s. The frequency stability noise floor of 100 MHz is 2.6 × 10^−15^ @ 1 s and 2.2 × 10^−16^ @ 100 s, and the frequency stability noise floor of 10 MHz is 4.3 × 10^−15^ @ 1 s, 2.6 × 10^−16^ @ 100 s, and 6.2 × 10^−17^ @ 1000 s. The results indicate that the frequency stability of the 100 MHz signal decreases with a slope close to 1/$\sqrt{\tau }$, demonstrating that the system has essentially reached the circuit noise limit. However, the frequency stability of the 10 MHz signal is noticeably worse than that of the 100 MHz signal. The primary reason for this difference is the additional noise introduced by the frequency divider when down-converting from 100 MHz to 10 MHz. Figure 4b shows the phase noise results of the 100 MHz and 10 MHz signals at different offset frequencies. The phase noise of 100 MHz is −117 dBc/Hz @ 100 Hz, −133 dBc/Hz @ 1 kHz, −157 dBc/Hz @10 kHz, −154 dBc/Hz @ 100 kHz, and −158 dBc/Hz @ 1 MHz. The phase noise of 10 MHz is −124 dBc/Hz@100 Hz, −143 dBc/Hz@1 kHz, −169 dBc/Hz @ 10 kHz (reaching the noise floor of 5125A), −169 dBc/Hz @ 100 kHz, and −166 dBc/Hz @ 1 MHz. The phase noise floor of 100 MHz is −154 dBc/Hz@100 Hz, −165 dBc/Hz @ 1 kHz, −169 dBc/Hz @ 10 kHz, and −169 dBc/Hz @ 1 MHz. The phase noise floor of 10 MHz is −167 dBc/Hz @ 100 Hz, −173 dBc/Hz @ 1 kHz, −175 dBc/Hz @ 10 kHz, and −173 dBc/Hz @ 1 MHz.

Currently, the best performance for chip optical clocks in terms of short-term stability is 4.4 × 10^−12^/$\sqrt{\tau }$ [26]. If the signal from such an optical clock is down-converted to the MM-wave range using a micro-cavity optical comb, the stability of the MM-wave signal would not exceed that of the optical clock. The down-conversion system reported in this paper can nearly eliminate any additional noise when down-converting the chip optical clock signal to classical time–frequency signals, thereby expanding the application potential of chip optical clocks.

## 4. Conclusions

This paper presents the design and implementation of a MM-wave down-conversion system based on low-noise regenerative frequency division technology, which successfully down-converts a 100 GHz MM-wave signal to 10 MHz and 100 MHz with low additional phase noise. Performance was validated through comparison experiments using two identical systems. The results show that the system achieves excellent frequency stability at 5.0 × 10⁻^15^ @ 1 s, 7.1 × 10⁻^15^ @ 100 s, and 1.8 × 10⁻^16^ @ 1000 s for the 100 MHz signal, along with low phase noise of −117 dBc/Hz @ 100 Hz and −157 dBc/Hz @ 10 kHz. For the 10 MHz signal, the system achieves even lower phase noise at −124 dBc/Hz @ 100 Hz and −169 dBc/Hz @ 10 kHz, along with high stability at 5.7 × 10⁻^14^ @ 1 s, 2.1 × 10⁻^15^ @ 100 s, and 5.9 × 10^−16^ @ 1000 s. Compared with existing down-conversion solutions, this study demonstrates significant improvements in noise suppression, frequency stability, and system robustness while reducing reliance on input signal frequency and optical path alignment. This provides a novel solution for frequency conversion and compatibility in high-precision optical clocks. The system’s performance not only meets current demands in the optical clock domain, but also significantly expands its potential applications in high-precision timekeeping, quantum communication, and navigation. However, the system’s scalability to higher frequency ranges and performance under dynamic conditions require further investigation. Future work will focus on extending the frequency range, achieving chip-level integration, and optimizing performance in dynamic environments.

## Data Availability

Restrictions apply to the availability of these data. The ownership of data belongs to the National Time Service Center (NTSC), Chinese Academy of Sciences, these data can be available from the corresponding author with the permission of NTSC.
